# Bipolar Disorder in a Young Girl with Dandy-Walker Syndrome

**Published:** 2015-06

**Authors:** Mahin Eslami Shahre Babaki, Fariborz Estilaee

**Affiliations:** 1 Neurology Research Center AND Department of Psychiatry, Afzalipour School of Medicine, Shahid Beheshti Hospital, Kerman University of Medical Sciences, Kerman, Iran; 2 Department of Psychiatry, Neyshabur University of Medical Sciences, Neyshabur, Iran

Dandy-Walker syndrome is a congenital brain malformation involving the cerebellum and the fourth ventricle. The key features of this syndrome are mental retardation, cerebellar ataxia, and symptoms related to hydrocephaly. The psychiatric aspects of this syndrome have been insufficiently appreciated. Described here is a 17- year- old girl with an acute manic episode emerged in the course of Dandy-Walker syndrome. Presentation and treatment are then addressed.

Historically, there has been a trend toward dichotomy of organic or nonorganic mental disorders. However, further insight into these disorders has made this categorization less valid. Currently, it is believed that virtually all organic brain syndromes show psychiatric symptoms in their courses; and on the other hand, mental disorders are perceived as having an organic, while not fully appreciated, base.

 Dandy-Walker syndrome is a congenital brain malformation involving the cerebellum and the fourth ventricle (1). It is occurred during the fourth and fifth weeks of pregnancy and is frequently associated with malformations of cardiovascular, musculoskeletal, urogenital and gastrointestinal systems (2).

The major presentations are mental disorder, cerebellar ataxia and symptoms related to hydrocephaly (3). Like other brain anomalies, it can be associated with psychiatric symptoms. This presentation is less prevalent, and it has been insufficiently discussed. Here, we are presenting a case with Dandy-Walker syndrome who was admitted into a psychiatric hospital.

## Material and Methods (case presentation)

The subject is a 17- year- old girl suffering from mental retardation, being referred first to a psychiatric hospital for her mental problems. Her signs started three months ago when she was considered as mostly walking during the night and having a decreased need to sleep. She used to be irritated whenever she could not communicate her demands. However, she started to show unprovoked anger. Her appetite increased as well. While not being able to speak completely, she was found to be getting more talkative than the past; she particularly talked to herself more.

 She went out of home and walked without any aim for hours. 

In this period, she did not have symptoms of paranoia, grandiosity or other delusions and did not develop any obsession or compulsion.

No problem had taken into consideration before she was born, but her developmental milestones showed a delay. She had started walking when she was three and she gradually began speaking at five. However, she could never speak more than a few words and a few short sentences. She was perceived as having some limitations in explaining her needs and she was dependent on others to take a bath and wear clothes. She did not have urinary or fecal incontinence.

Also, no symptoms of stereotypic movement, attention deficit and hyperactivity have been found in her past psychiatric history.

Although she had have behavior disturbance and occasional aggression and self-biting, psychiatric management was not done for her.

There were not any medical or psychiatric disorders in her family history, and her sister and brother were normal.

In terms of physical examination, she was relatively thin and showed bradykinesia and mild generalized rigidity. She did not have tremor or any obvious sign of physical abnormality. With regards to mental state examination, she had no eye contact, and her mood was considered as irritable. She showed echolalia and poverty of speech. As a result, her thought and perception could not be evaluated. She was not able to take the IQ test either. However, based on the history and clinical judgment, she was diagnosed as having deep mental retardation.

She was admitted to hospital with the diagnosis of bipolar I disorder (the most recent episode: mania) in axis I and deep MR in axis II. In her brain MRI, enlargement of fourth ventricle and cisterna magna and cerebellar vermis atrophy were seen. EEG showed unspecific changes and there was no positive finding in her blood count, electrolytes, renal and liver function tests and blood sugar. 

Despite two weeks of treatment with risperidone 3 mg/day and biperidine 4 mg/day and partial control of some symptoms, irritability and anger remained consistently. Therefore, valproic acid (VPA) 400 mg/ day was added to the treatment and after one month the patient was discharged from the hospital with complete remission of newly presented symptoms.

## Discussion

Dandy-Walker syndrome is regarded as a relatively rare brain disorder. Sometimes, it is diagnosed early after birth (4), but sometimes it remains undiagnosed until the later childhood or early adulthood; being shown as a mental retardation, it might be discovered incidentally (5, 6). The psychiatric aspect of this syndrome has not been addressed sufficiently. To our knowledge, there are few studies of psychiatric presentation of this syndrome (3, 7, and 8).

This report presents a 17- year- old MR girl whose reason of admission was a manic episode. Considering the clinical examination and imaging of the case, Dandy- Walker syndrome was diagnosed. This report indicates the importance of taking psychiatric symptoms into consideration while evaluating organic brain disorders. With respect to treatment, the same treatment as the primary mood disorder was indicated effective. However, like other brain disorders, lower doses should be used as these patients are more sensitive to side effects of psychotropic drugs.

Finally, it seems that it is too soon to arrive at a conclusion about whether dandy walker syndrome can be a risk factor or precipitate affective disorders such as bipolar disorder.

Another assumption which calls for more research is the common genetics factor between dandy walker syndrome and bipolar disorder.
